# MEK1 Inhibits Cardiac PPARα Activity by Direct Interaction and Prevents Its Nuclear Localization

**DOI:** 10.1371/journal.pone.0036799

**Published:** 2012-06-19

**Authors:** Hamid el Azzouzi, Stefanos Leptidis, Meriem Bourajjaj, Marc van Bilsen, Paula A. da Costa Martins, Leon J. De Windt

**Affiliations:** 1 Interuniversity Cardiology Institute Netherlands, Royal Netherlands Academy of Sciences, Utrecht, The Netherlands; 2 Department of Cardiology, Cardiovascular Research Institute Maastricht, Maastricht University, Maastricht, The Netherlands; 3 Department of Physiology, Cardiovascular Research Institute Maastricht, Maastricht University, Maastricht, The Netherlands; Harvard Medical School, United States of America

## Abstract

**Background:**

The response of the postnatal heart to growth and stress stimuli includes activation of a network of signal transduction cascades, including the stress activated protein kinases such as p38 mitogen-activated protein kinase (MAPK), c-Jun NH2-terminal kinase (JNK) and the extracellular signal-regulated kinase (ERK1/2) pathways. In response to increased workload, the mitogen-activated protein kinase kinase (MAPKK) MEK1 has been shown to be active. Studies embarking on mitogen-activated protein kinase (MAPK) signaling cascades in the heart have indicated peroxisome-proliferators activated-receptors (PPARs) as downstream effectors that can be regulated by this signaling cascade. Despite the importance of PPARα in controlling cardiac metabolism, little is known about the relationship between MAPK signaling and cardiac PPARα signaling.

**Methodology/Principal Finding:**

Using co-immunoprecipitation and immunofluorescence approaches we show a complex formation of PPARα with MEK1 and not with ERK1/2. Binding of PPARα to MEK1 is mediated via a LXXLL motif and results in translocation from the nucleus towards the cytoplasm, hereby disabling the transcriptional activity of PPARα. Mice subjected to voluntary running-wheel exercise showed increased cardiac MEK1 activation and complex formation with PPARα, subsequently resulting in reduced PPARα activity. Inhibition of MEK1, using U0126, blunted this effect.

**Conclusion:**

Here we show that activation of the MEK1-ERK1/2 pathway leads to specific inhibition of PPARα transcriptional activity. Furthermore we show that this inhibitory effect is mediated by MEK1, and not by its downstream effector kinase ERK1/2, through a mechanism involving direct binding to PPARα and subsequent stimulation of PPARα export from the nucleus.

## Introduction

Peroxisome proliferator-activated receptor α (PPARα) is a nuclear receptor which is believed to act as a sensor of fatty acids (FA) and FA derivatives to enable the cell to adapt to environmental changes through regulation of a large number of processes such as inflammation, differentiation and metabolism [1]. PPARα is expressed in metabolically active tissues including the liver, brown fat, kidney, skeletal muscle and heart [2]. Transgenic mice with forced overexpression of PPARα in cardiac muscle display increased FA oxidation rates, accumulation of triacylglycerides, decrease in glucose metabolism and eventually develop cardiomyopathy [3], [4]. In turn, mice deficient for PPARα have elevated plasma FA levels as a consequence of inadequate FA oxidation, rendering them hypoglycemic as a result of their reliance on glucose [5]. The natural ligands for PPARα are long-chain FA and several eicosanoids. Synthetic ligands for the PPARs comprise hypolipidemic, anti-inflammatory and insulin-sensitizing drugs. In the presence of a ligand, PPARs adopt an active conformation by forming an obligate heterodimer with the retinoid X receptor (RXR). Recruitment of additional co-activators leads to binding to peroxisome proliferator response elements (PPRE) in target genes, provoking PPAR-dependent gene expression.

During the development of cardiac hypertrophy, myocardial fatty acid oxidation (FAO) rates decrease and glucose utilization increases [6]. During this transition, the reduced nuclear level of PPARα suggests that this phenomenon may be responsible for downregulation of cardiac FAO genes in the hypertrophied heart [7]. Hence, understanding the mechanisms that regulate the activity of PPARα is crucial to determine the precise contribution of altered FAO at the genesis and progression to heart failure. Apart from the classical ligand-dependant regulation, several studies have reported the modulation of PPARα activity by phosphorylation. For example, insulin treatment induces phosphorylation, at the serine residues 12 and 21 in the transactivation domain, and subsequent activation of PPARα [8]. PKA activators have also been shown to modulate the activity of PPARα through phosphorylation of several sub domains, including the DNA-binding domain and the ligand binding domain [9].

The response of the postnatal heart to growth and stress stimuli includes activation of a network of signal transduction cascades, including the stress activated protein kinases such as p38 mitogen-activated protein kinase (MAPK), c-Jun NH_2_-terminal kinase (JNK) and the extracellular signal-regulated kinase (ERK1/2) pathways [10]. Studies embarking on the regulation of PPARα activity in cardiac muscle have indicated PPARα as a downstream effector of MAPK signaling [11]. In line, the MEK1-ERK1/2 pathway was shown to inhibit PPARγ transcriptional activity in non-cardiac cells [12]. Moreover, members of the p38 kinase family have been shown to phosphorylate PPARα in ligand-dependent manner, resulting in enhanced transcriptional activity [11]. Transgenic mice with cardiac-restricted expression of an activated form of MEK1 developed a physiological concentric hypertrophy response with preserved cardiac function [13], indicating an important role for MEK1-ERK1/2 signaling pathway during forms of cardiac hypertrophy. It is interesting to note that activation of the MEK1-ERK1/2 pathway led to inhibition of PPARγ transcriptional activity in non-cardiac cells [12], [14], indicating a diverse effect of the different MAPK pathways on PPAR activity. Despite the importance of PPARα on FAO and cardiac metabolism, little is known about the relationship between MEK1-ERK1/2 pathway and cardiac PPARα signaling. Here we show that activation of the MEK1 pathway leads to inhibition of cardiac PPARα transcriptional activity. Furthermore we show that the inhibitory effect is mediated by MEK1 rather than MEK1-ERK1/2 phosphorylation events, through a mechanism involving interaction of MEK1 with PPARα and subsequent nuclear export of PPARα.

## Results

### MEK1 Inhibits PPARα Activity in an ERK1/2-independent Fashion

**Figure 1:**
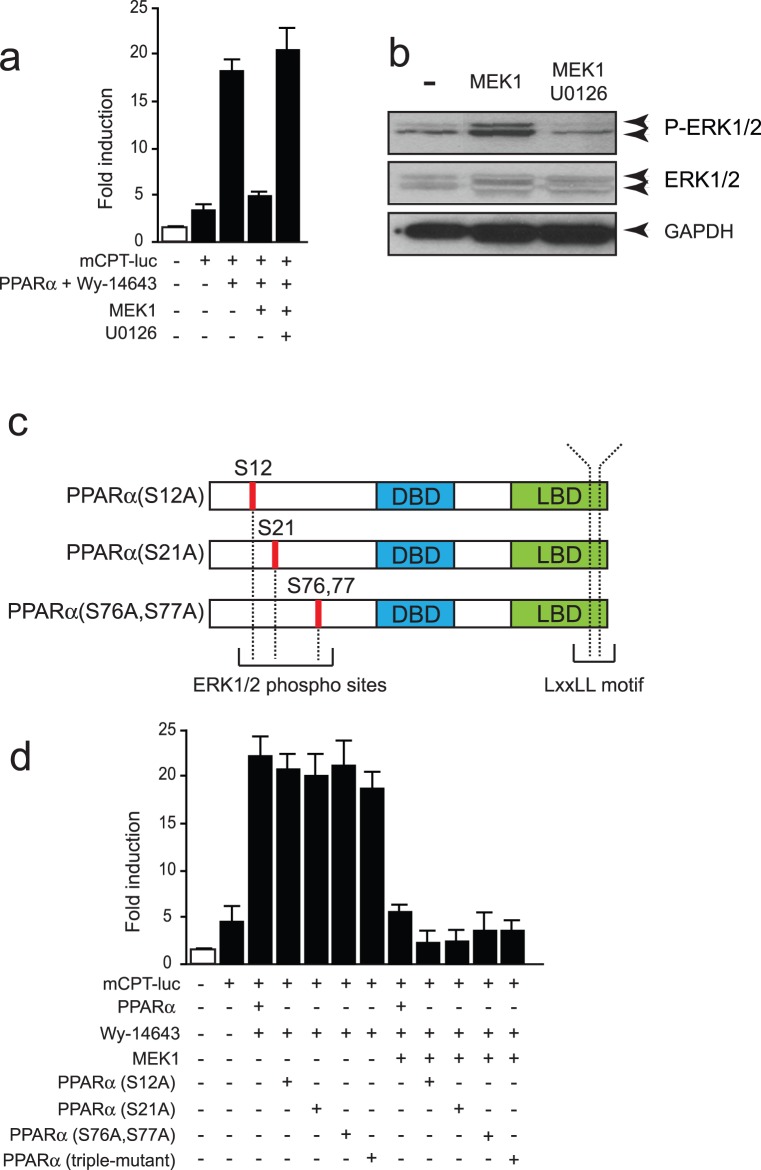
MEK1 expression inhibits PPARα transcriptional activity in an ERK1/2 phosphorylation independent manner. (a) Luciferase measurements on NkL-Tag cells transiently transfected with a *mCPT* promoter driven reporter as a functional readout for PPARα activity after co-transfection with PPARα-V5 and MEK1 for 24 hr, and stimulated with Wy-14643 (1 µM) or U0126 (5 µM) as indicated, for 2 hours. (b) Western blot analysis using anti-phosphorylated ERK1/2 (p-ERK1/2) antibody on lysates of NkL-Tag cells transiently transfected with MEK1 for 24 hr, indicating enhanced activation of ERK1/2 after MEK1 expression. (c) Schematic representation of the *trans*-activating domain of PPARα along with the three putative phosphorylation target sites for ERK1/2 and the LXXLL motif. (d) Luciferase measurements of NkL-Tag cells transiently transfected with a *mCPT* promoter driven reporter and co-transfected with mutants of PPARα-V5 and MEK1 for 24 hr, and stimulated 2 hours with Wy-14643 (1 µM), indicating MEK1 induced inhibition of PPARα to be ERK1/2 phosphorylation-independent. pGL3-luc construct was transiently transfected as a negative control (white bar).

It has been shown that phosphorylation of PPARs can attenuate their transcriptional activity in a sub-type specific way. Given the proven importance of PPARα [15] and the MEK1-ERK1/2 pathway [13], [16], [17] on cardiac muscle viability and hypertrophy, we studied the effect of the MEK1-ERK1/2 cascade on PPARα activity in detail by examining the transcriptional activity of PPARα in the presence of exogenous MEK1 constructs or their inhibitors. To this end, we resorted to the use of a previously developed, ventricular muscle cell line, NKL-TAg. NKL-TAg cells actively proliferate without apparent senescence, while introduction of Cre recombinase results in the elimination of TAg expression, permanent exit from the cell cycle and expression of cardiac markers [18]. As a functional verification of the transcriptional activity of PPARα, we performed transient transfection of a luciferase reporter driven by a *cpt1a* promoter harboring a functional PPRE. Co-transfection of a PPARα expression vector and stimulation with Wy-14643 as synthethic ligand (2 hr) resulted in increased luciferase activity (Fig. 1a), indicating enhanced transcriptional activity of PPARα. This effect was completely abrogated when MEK1 was co-expressed (Fig. 1a). Addition of U0126, a specific inhibitor of MEK1, re-activated Wy-14643 mediated PPARα induction of the mCPT-luc reporter, indicating that the inhibitory effect involved MEK1 activation.

A classical downstream effector of MEK1 is ERK1/2. Western Blot analysis showed increased phosphorylation of ERK1/2 after MEK1 activation. Moreover, addition of U0126 inhibited MEK1 induced phosphorylation of ERK1/2 (Fig. 1b), indicating the efficiency of used expression vectors and efficiency of U0126.

Previous studies indicated that, in addition to the C-terminal ligand-binding AF2 domain, the N-terminal *trans*-activating AF1 domain plays an important role in the regulation of the transcriptional activity of PPARα [19]. Analysis of the AF1 domain, amino acids 1–92, of PPARα revealed three putative phosphorylation target sites for ERK1/2 (Fig. 1c). To test whether ERK1/2-mediated phosphorylation of one or more putative phospho-acceptor sites on PPARα may underlie the MEK1-ERK1/2-mediated inhibition of PPARα transcriptional activity, we employed site-directed mutagenesis to create different PPARα constructs harboring a single or multiple serine to alanine conversion at the indicated amino acid residues (Fig. 1c). Surprisingly, MEK1 induced inhibition was not hampered by co-transfection of the single mutated PPARα constructs (Fig. 1d). Furthermore, co-transfection of a PPARα construct harboring a mutation at three putative phosphorylation target sites for ERK1/2 (Triple-muta) did not change the MEK1 induced inhibition (Fig. 1d).

**Figure 2:**
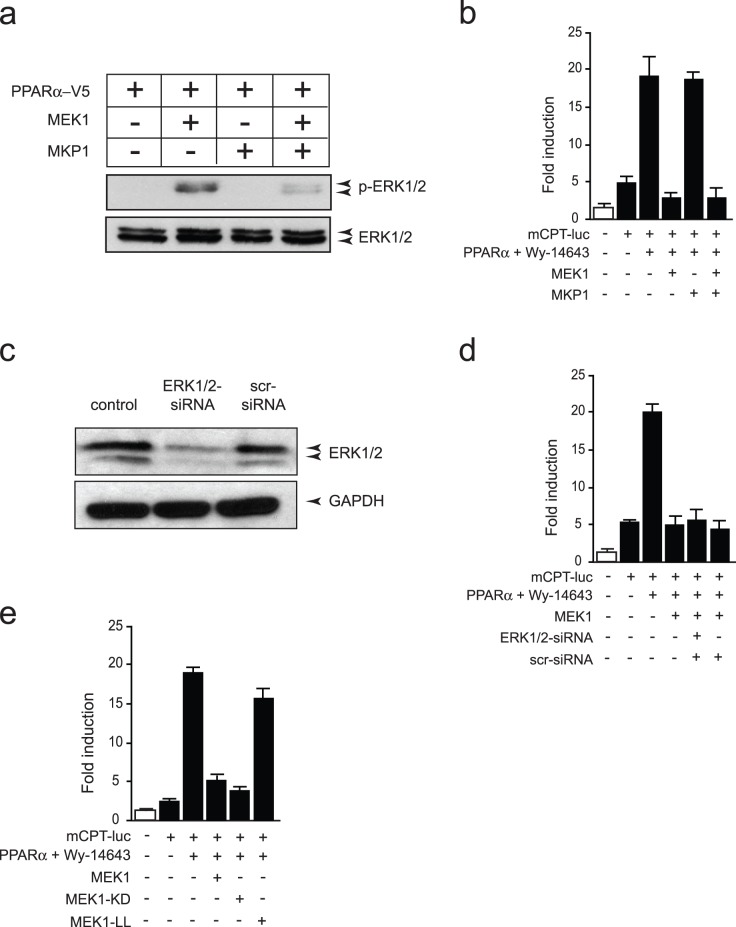
Inhibition of PPARα by MEK1 relies on the nuclear export of MEK1 and not on MEK1 kinase activity. (a) Western blot analysis using anti-phosphorylated ERK1/2 (p-ERK1/2) antibody on lysates of NkL-Tag cells transiently transfected with MEK1 and MKP1, indicating decreased activation of ERK1/2 after co-expression of MKP1. (b) Luciferase measurements of NkL-Tag cells transiently transfected with a *mCPT* promoter driven reporter and co-transfected with PPARα-V5, MEK1 and MKP1, and stimulated 2 hours with Wy-14643 (1 µM) as indicated. (c) Western blot analysis using anti-ERK1/2 (ERK1/2) antibody on lysates of NkL-Tag cells transiently transfected with siRNAs against ERK1 and ERK2, or scrambled siRNA as a negative control (scr), indicating decreased levels of ERK1/2 after co-transfection of siRNAs targeting ERK1/2. (d) Luciferase measurements of NkL-Tag cells transiently transfected with a *mCPT* promoter driven reporter and co-transfected with PPARα-V5, MEK1 and siRNAs, and stimulated 2 hours with Wy-14643 (1 µM), as indicated. (e) Luciferase measurements of NkL-Tag cells transiently transfected with a *mCPT* promoter driven reporter and co-transfected with PPARα-V5, MEK1, MEK1-KD and MEK1-LL, indicating that the inhibition of PPARα by MEK1 relies on the nuclear export of MEK1 and not on MEK1 kinase activity. pGL3-luc construct was transiently transfected as a negative control (white bar).

As a second approach to exclude direct ERK1/2 phosphorylation events on PPARα, we set up an assay using the MAPK phosphatase MKP1, which provokes ERK1/2 dephosphorylation, to confirm that the MEK1 inhibitory effect is unrelated to activation of ERK1/2. As indicated by Western Blot, co-transfection of MKP1 led to dephosphorylation of ERK1/2 even in the presence of activated MEK1 (Fig. 2a). Interestingly, dephosphorylation of ERK1/2, by transient co-transfection of MKP1, did not change the inhibitory effect of MEK1 on the transcriptional activity of PPARα (Fig. 2b). In concordance, siRNA based knockdown of ERK1/2 (Fig. 2c) did not impair the reduction of transcriptional activity of PPARα by MEK1 (Fig. 2d). To verify whether the MEK1 induced inhibition of PPARα transcriptional activity is not related to the kinase activity of MEK1, transient co-transfection of a kinase inactive MEK1 (MEK1-KD) did not change the PPARα transcriptional activity inhibition induced by MEK1 (Fig. 2e), suggesting an unlikely role for phosphorylation induced by MEK1. On the other hand, using a MEK1 mutant lacking the nuclear localization signal (MEK1-LL) significantly restored PPARα transcriptional activity (Fig. 2e). Taken together, these findings indicate a minor role for ERK1/2 or MEK1 activity on the putative phospho-acceptor sites in the trans-activating domain of PPARα as a mechanism responsible for MEK1-ERK1/2 mediated inhibition of PPARα transcriptional activity, but likely to be mediated by MEK1 subcellular localization.

### MEK1 Binding to PPARα Provokes Nuclear Export

**Figure 3:**
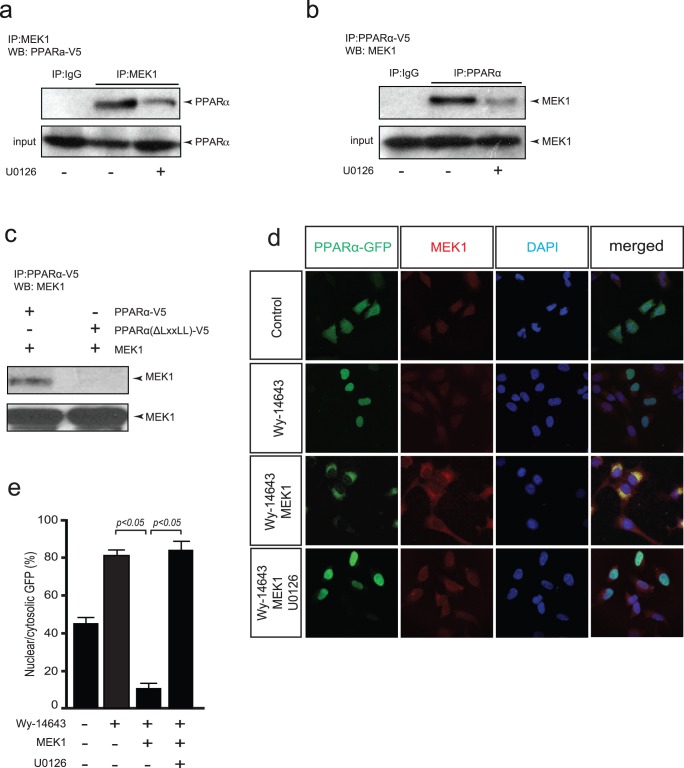
MEK1 interaction with PPARα induces nuclear export. (a) Western blot analysis on precipitates of HEK293 cells transiently co-transfected with PPARα-V5, MEK1 and treated with U0126 (5 µM) or not for 2 hr and immunoprecipitated using an anti-MEK1 antibody. (b) Western blot analysis on precipitates of HEK293 cells transiently co-transfected with PPARα-V5, MEK1 and treated with U0126 (5 µM) as indicated for 2 hr and co-immunoprecipitated using an anti- PPARα antibody. (c) Western blot analysis on precipitates of HEK293 cells transiently transfected with a mutant PPARα-GFPΔ(LxxLL)-V5 expression vector (lacking the LxxLL motif) with or without a MEK1 expression vector and stimulation with Wy-14643 (1 µM) for 2 hr, and immunoprecipitated using an anti-PPARα antibody. (d) Fluorescence immunocytochemistry images of HEK293 cells transiently co-transfected with a PPARα-GFP expression vector, with or without a MEK1 expression vector and stimulation with or without Wy-14643 for 2 hr (1 µM), showing co- cytoplasmic translocation of PPARα and co-localization with MEK1 after co-transfection with MEK1. Addition of U0126 (5 µM) inhibited the MEK1 induced translocation (lower panels). (e) Bar graph indicates mean ± SEM of the percentage of nuclear GFP, showing decreased nuclear PPARα-GFP after co-transfection with MEK1. Addition of U0126 inhibited the MEK1 induced translocation of PPARα-GFP.

Since the MEK1 inhibitory effect on PPARα activity could not be explained through the classical MEK1-ERK1/2 pathway, we examined whether the inhibitory effect of MEK1 on PPARα activity is mediated by direct protein-protein interaction. HEK-293 cells were co-transfected with PPARα and MEK1 expression vectors. After 2 hr of stimulation with the synthethic ligand Wy-14643, cells were subjected to co-immunoprecipitation using a MEK1 antibody, resulting in efficient precipitation of PPARα (Fig. 3a). Interestingly, co-stimulation with the specific MEK1 inhibitor, U0126, impaired immunoprecipitation of PPARα by MEK1 (Fig. 3a). Using the same conditions, we repeated the experiments using a PPARα antibody to co-immunoprecipitate MEK1. Also under these conditions, MEK1 was readily immunoprecipitated, indicating an interaction between PPARα and MEK1, while co-stimulation with U0126 resulted in reduced levels of precipitated MEK1 (Fig. 3b). IgG was used as a negative control for the specificity of the antibodies used for the coimmunoprecipitation of PPARα or MEK1. Importantly, when we used a PPARβ/δ expression vector, no co-immunoprecipitation of MEK1 was observed (data not shown), indicating the specificity of the interaction between MEK1 and PPARα.

Protein-protein interactions with MEK1 are often mediated by the CRS/CD domain, which allows interaction with other regulatory proteins. Within the MEK1 CRS/CD domain interaction has been shown to be mediated by an LXXLL binding pocket in binding partners for MEK1 [20]. Characterization of the ligand-binding domain of PPARα revealed an LXXLL binding pocket (Fig. 1c). We constructed a V5-tagged PPARα expression vector harboring a truncation of the last 16 amino acids of the c-terminal AF2 domain, lacking the LXXLL binding pocket. Using this construct, co-immunoprecipitation with MEK1 failed (Fig. 3c), while immunoprecipitation between MEK1 and wild-type PPARα was efficient, indicating that this motif is a crucial structural element for the protein-protein interaction between MEK1 and PPARα.

Since MEK1 was shown not to directly phosphorylate PPARγ [12], we next considered subcellular localization as an important factor participating in the regulation of PPAR signaling [21]. We therefore investigated whether the direct interaction serves as a new mechanism for regulating the subcellular localization of PPARα. To this end, HEK293 cells were transiently co-transfected with an expression vector containing a PPARα -GFP fusion with or without a MEK1 expression vector and stimulation with Wy-14643 (2 hr). Cells were then fixed and PPARα localization was determined by GFP fluorescence while MEK1 was immunostained using a MEK1 antibody. PPARα-GFP remained largely in the cytosol and massive nuclear translocation was induced only after stimulation with Wy-14643 (Fig. 3d). In contrast, ectopic expression of an activated form of MEK1 colocalized with PPARα resulting in a massive exclusion of PPARα from the nucleus towards a predominant cytoplasmic localization (Fig. 3d). Addition of U0126 inhibited the MEK1 induced translocation, causing PPARα-GFP to be localized in the nucleus after stimulation with Wy-14643 (Fig. 3d). Quantification of nuclear PPARα-GFP indicated a significant translocation towards the cytosol after ectopic expression of an activated form of MEK1, which was repressed after the addition of U0126 (Fig. 3d). Taken together, these findings reveal a novel mechanism for the regulation of cardiac PPARα activity via interaction and subsequent inactivation by MEK1.

### MEK1 Interacts with the LXXLL Binding Pocket of PPARα and not of PPARβ/δ

**Figure 4:**
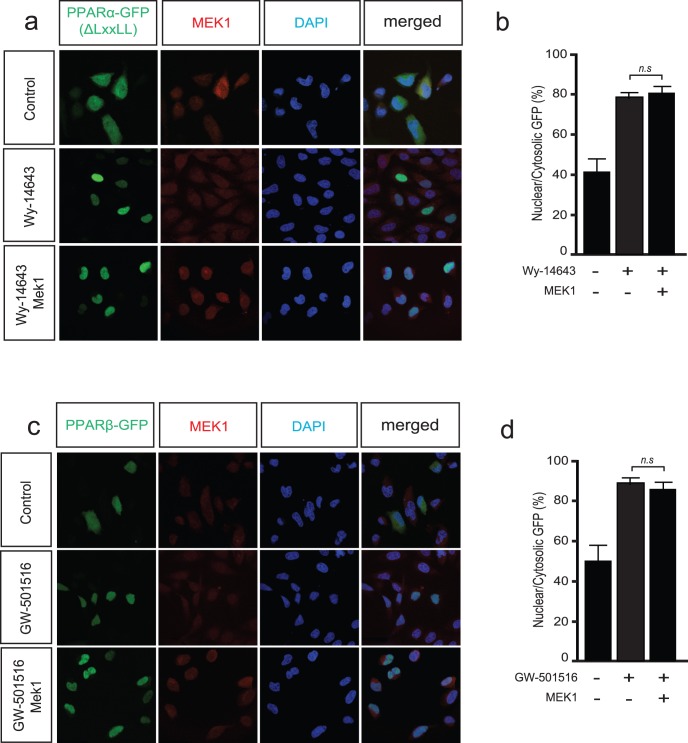
MEK1 interacts with PPARα via the LxxLL motif. (a) Fluorescence immunocytochemistry images of HEK293 cells transiently co-transfected with a mutant PPARα-GFPΔLxxLL (lacking the LxxLL motif) with or without a MEK1 expression vector and stimulation with or without Wy-14643 (1 µM) for 2 hr. (b) Bar graph indicates mean ± SEM of the percentage of nuclear GFP, showing no significant changes in nuclear PPARα-GFPΔLxxLL after co-transfection with MEK1. (c) Fluorescence immunocytochemistry images of HEK293 cells transiently co-transfected with a PPARβ/δ-GFP with or without a MEK1 expression vector and stimulation with or without the PPARβ/δ-selective agonist GW-510516 (1 µM) for 2 hr. (d) Bar graph indicates mean ± SEM of the percentage of nuclear GFP, showing no significant changes in nuclear PPARβ/δ-GFP after co-transfection with MEK1, indicating that MEK1 does not interact with this PPAR isoform.

In light of our earlier results, we reasoned that MEK1-induced PPARα translocation was mediated through direct interaction of MEK1 with the LXXLL binding pocket of PPARα. We therefore designed a PPARα-GFP construct that harbored a truncation of the last 16 amino acids of the C-terminal AF2 domain of PPARα, lacking the necessary LXXLL binding pocket (PPARα-GFPΔLxxLL). Next, we repeated the experiment using PPARα-GFPΔLxxLL and analyzed subcellular localization in the presence of absence of MEK1 and Wy-14643. In this case, Wy-14643 induced nuclear localization of PPARα-GFPΔLxxLL was not affected by ectopic MEK1 expression (Fig. 4a). Quantification of nuclear PPARα-GFPΔLxxLL indicated a significant translocation towards the nucleus after stimulation with Wy-14643 that was not altered after ectopic expression of an activated form of MEK1 (Fig. 4b). Co-transfection of MEK1 with PPARβ/δ-GFP did not result in nuclear extrusion, even though this construct also contains a LXXLL element (Fig. 4c,d).

In conclusion, the combined experiments demonstrate that the ERK1/2 selective MAPKK MEK1 interacts with the LXXLL binding pocket of PPARα and forces translocation out of the nucleus to the cytosol as a novel mechanism whereby MEK1 signaling inhibits the transcriptional activity of PPARα.

### MEK1 Activation Attenuates PPARα Transcriptional Activity in vivo

**Figure 5:**
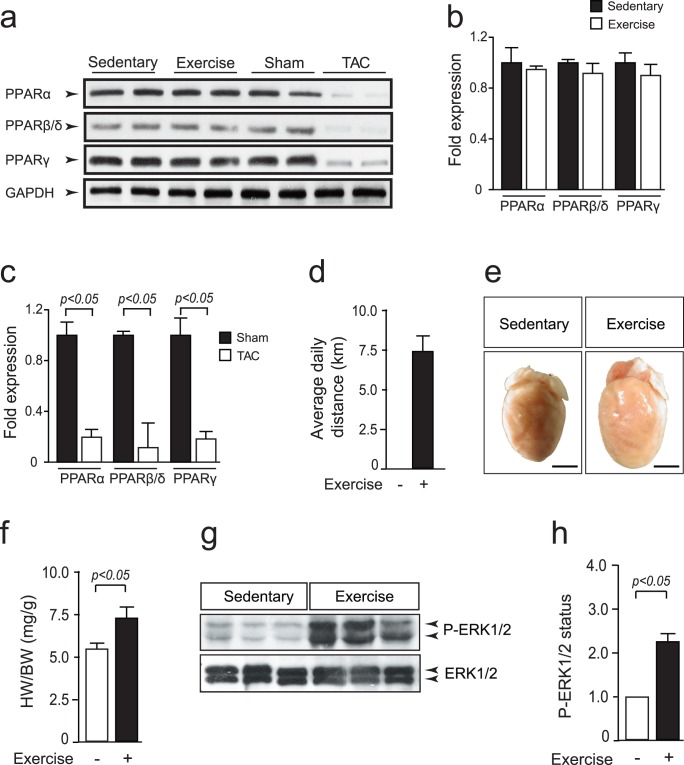
Voluntary running-wheel exercise stimulates cardiac MEK1 activation. (a) Western blot analysis using anti-PPAR antibodies on lysates of heart samples of indicated experimental procedure, showing reduced PPAR expression of mice hearts subjected to transverse aortic constriction (TAC). (b) Quantification of PPAR protein levels of in hearts from sedentary or exercised mice (n = 6 per group). (c) Quantification of PPAR protein levels of in hearts from sham or transverse aortic constricted mice ( = 6 per group). (d) Average daily distance that mice ran voluntarily. (e) Representative images of hearts from mice that remained sedentary or were subjected to voluntary wheel exercise for 4 weeks. Note the increase in size of the exercised heart. (f) Heart weight to body weight (HW/BW) ratios of mice that remained sedentary or were subjected to voluntary wheel exercise (n = 8 per group). (g) Western blot analysis using anti-phosphorylated ERK1/2 (p-ERK1/2) antibody on lysates of indicated heart samples, demonstrating enhanced MEK1-ERK1/2 activity following exercise-induced cardiac hypertrophy. (h) Quantification of the phosphorylation status of ERK1/2 in hearts from sedentary or exercised mice (n = 6 per group).

*In vivo*, MEK1 is activated in hearts of mice subjected to exercise training [22], indicating the association of MEK1 with cardiac adaptation to increased workload. By Western blotting analysis, we verified that all PPAR isoforms maintained similar expression levels following exercise training (Fig. 5a, b). In contrast, Western blotting analysis showed severely decreased PPARα levels in hearts of mice subjected to pressure overload, indicating different regulatory mechanisms of PPARα activity after increased cardiac workload due to pressure overload (Fig. 5a, c). To determine whether the discovered mechanism of MEK1 mediated PPARα inhibition also occurred *in vivo*, we chose voluntary running-wheel exercise as a model to stimulate physiological cardiac hypertrophy and activate cardiac MEK1 in mice. After 4 weeks of voluntary wheel exercise, mice demonstrated a substantial cardiac growth response as evidenced by increased HW/BW ratios (Fig. 5d, e, f). MEK1 activation was verified by analyzing cardiac lysates for phospho-ERK1/2 status (Fig. 5g, h).

**Figure 6:**
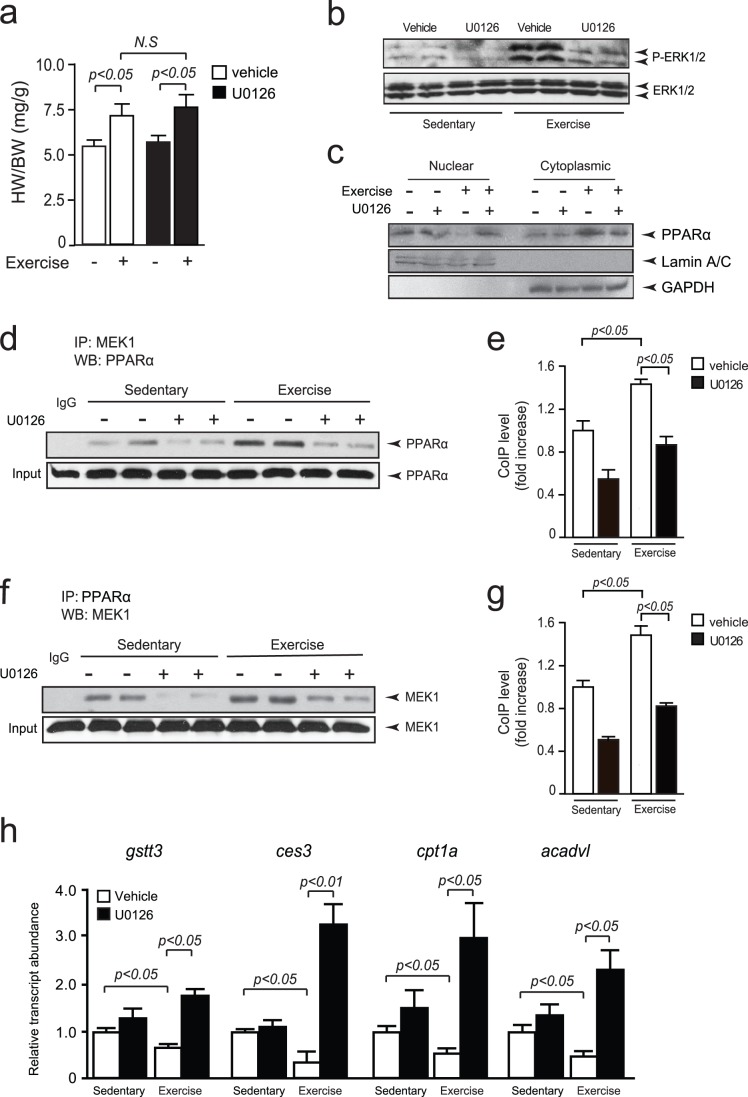
Activation of MEK1 during physiological cardiac hypertrophy inhibits PPARα activity. (a) Heart weight to body weight (HW/BW) ratios of mice treated with vehicle or U0126, and subjected to voluntary wheel exercise or not (n = 8 per group). (b) Western blot analysis using anti-phosphorylated ERK1/2 (p-ERK1/2) antibody on lysates of heart samples of indicated experimental procedure, showing reduced MEK1-ERK1/2 activity of exercised mice treated with U0126. (c) Western blot analysis using anti-PPARα antibody on nuclear and cytosolic fractions of lysates of heart samples, indicating decreased nuclear PPARα levels and increased cytosolic PPARα levels following exercise-induced cardiac hypertrophy. Treatment with U0126 reduced the MEK1 induced cytoplasmic translocation of PPARα. (d) Western blot analysis using anti- PPARα antibody on precipitates of heart lysates of exercised or sedentary mice, treated with U0126 or vehicle and immunoprecipitated using an anti-MEK1 antibody. (e) Quantification of the co-immunoprecipitated PPAR protein levels in hearts from sedentary or exercised mice (n = 6 per group), treated with U0126 or vehicle. (f) Western blot analysis using anti-MEK1 antibody on precipitates of heart lysates of exercised or sedentary mice, treated with U0126 or vehicle and immunoprecipitated using an anti-PPARα antibody. (g) Quantification of the co-immunoprecipitated MEK1 protein levels in hearts from sedentary or exercised mice, treated with U0126 or vehicle (n = 6 per group). (h) RT-PCR analyses of PPARα target genes expression in sedentary and exercised hearts, treated with vehicle or U0126, indicating decreased PPARα activity during physiological hypertrophy.

Cohorts of wild-type mice were treated with either vehicle or U0126 and subjected to voluntary wheel exercise. U0126 did not affect exercise induced physiological cardiac hypertrophy (Fig. 6a), but U0126 efficiently prevented MEK1-ERK1/2 activation following exercise (Fig. 6b). To confirm the previous *in vitro* results, nuclear and cytosolic fractionation of these experimental heart lysates indicated decreased nuclear and increased cytosolic PPARα levels in exercised mice compared to sedentary animals (Fig. 6c). In contrast, treatment with U0126 increased nuclear localization of PPARα following exercise-induced activation of MEK1 (Fig. 6c). Next, heart lysates were subjected to immunoprecipitation assays using a MEK1 antibody. PPARα precipitation was accomplished in exercised mice treated with vehicle, while mice treated with U0126 showed significantly decreased PPARα precipitation in exercised animals (Fig. 6d, e). Conversely, immunoprecipitation with a PPARα antibody also efficiently precipitated endogenous MEK1 in exercised mouse hearts, while U0126 treatment resulted in significantly reduced levels of precipitated MEK1 (Fig. 6f.g).

Finally, we analyzed the expression of specific PPARα target genes [23] as a surrogate for functional PPARα activity in sedentary and exercised mouse hearts. In line with our expectation, MEK1 activation *in vivo* resulted in downregulation of transcripts for the PPARα target genes *glutathione s-transferase 3* (*gstt3*), *carboxylesterase 3 (ces3), carnitine o-palmitoyltransferase 1(cpt1a),* and *acyl-coenzyme a dehydrogenase (acadvl)*, while exercise in the presence of U0126 fully relieved the inhibition of these PPARα target genes (Fig. 6h). These findings support the notion that MEK1 signaling inhibits the transcriptional activity of PPARα and influences cardiac metabolic gene programs *in vivo*.

## Discussion

Mechanisms that regulate the ligand-independent activity of nuclear receptors, such as PPARs, are poorly understood and are often associated with kinase-dependent processes. Several consensus phosphorylation sites for PPARα have been identified including glycogen synthase kinase 3 (GSK3), protein kinase A (PKA), protein kinase C (PKC) and mitogen-activated protein kinase (MAPK). MAPK signaling pathways have been reported to be very important in the regulation of cellular differentiation, proliferation and stress responsiveness. Consisting of three major branches of sequentially signaling pathways, the MEK1 signaling pathway, which culminates in ERK1/2 activation, is hypothesized to regulate the growth and adaptation of the heart to both physiological and pathological stimuli [13].

Functional verification of the transcriptional activity of PPARα, after activation of the MEK1 pathway, resulted in a dramatic decrease of PPARα ability to activate a mCPT reporter. Although it was previously shown that activated ERK1/2 is able to phosphorylate PPARα [11], site-directed mutagenesis of all of the putative serine ERK1/2 phosphorylation target sites showed no difference to the MEK1 induced inhibition of PPARα transcriptional activity. Furthermore, inactivating ERK1/2 using the MAPK phosphatase MKP1 did not change the inhibitory effect of MEK1 on the transcriptional activity of PPARα indicating an insignificant role for ERK1/2. This also indicated that the MEK1 induced inhibition of PPARα is unlikely to be phosphorylation mediated since it was shown that PPARα is phosphorylated exclusively on serine residues *in vivo* with no detectable threonine or tyrosine activity [24]. In addition, MEK1 has been shown to be unable to phosphorylate PPAR [12].

An important factor that participates in the regulation of PPAR as well as MAPK signaling is their subcellular localization [21], [25]. Regarding the MAPK signaling, it has been shown that both ERK1/2 and MEK1 resides in the cytosol of resting cells and translocate into the nucleus upon stimulation [25]. However, while ERK1/2 has been shown to remain in the nucleus for a considerable time, MEK1 is quickly exported out of the nucleus due to its nuclear export signal (NES) [13], [26]. Furthermore, overexpression of active MEK1 interacts with PPARγ in the nucleus, allowing subsequent nuclear export of PPARγ [12]. Indeed, immunofluorescence experiments showed that ectopic expression of an activated form of MEK1 results in a significant translocation of PPARα-GFP towards the cytosol, which was abrogated after addition of U0126.

Unfortunately, little is known about the regulation of the intracellular distribution of PPARs, though cytosolic localization of PPARs has been reported as well as their bindings ability to the cytosolic/membrane proteins such as HSP90 [19], [20], [21], [27]. Interestingly, the presence of a CRS/CD domain in PPARα, which facilitates a protein-protein interaction with MEK1, indicated a possible interaction with MEK1. In line, ectopic addition of MEK1 resulted in a massive extrusion of PPARα, but not of PPARβ/δ, from the nucleus towards the cytoplasm even after stimulation with its synthetic ligand. Furthermore, truncation studies showed that interaction of PPARα with MEK1 is mediated via a LXXLL binding pocket, given that deletion of this motif resulted in loss of the inhibitory effect of MEK1. Although, co-immunoprecipitation studies showed a complex formation of PPARα with MEK1, but not with ERK1/2 (data not shown), it remains unknown whether this interaction is direct or indirect via unidentified additional components.

In this context one could assume that MEK1, via the inhibition of PPARα, has a role in the regulation of metabolic processes in the heart. Indeed, gene profiling studies executed on cardiomyocytes overexpressing a constitutively active form of MEK1 (MEK-EE) demonstrated a significant decrease of genes coding for proteins involved in FA metabolism [28]. These included genes that would be localized to the mitochondria and involved in FA translocation and oxidation (*cpt1a, acadv1, hadhsc*), but also binding proteins that are involved in cellular transport of FA (*cd36*). This effect was less apparent for the regulation of genes involved the glycolysis/gluconeogenesis, where glucose transporter GLUT3 was up regulated but other components of the glycolysis were downregulated. Nonetheless, this expression profile rendered cardiomyocytes more resistant to energy deprivation following deoxyglucose exposure [13], indicating a preserved intrinsic reserve. Additionally, analysis of cardiac substrate metabolism in PPARα knockout hearts indicated a substrate switch from FA to glucose and lactate but with an inability to respond to high energy demand, such as high workload, resulting in energetic and contractile failure mimicking end stage heart failure [29].

Gene expression studies performed with RNA isolated from ventricles of mice subjected to transverse aortic banding indicated a downregulation of PPARα expression and several of its target genes [30], [31]. Furthermore, protein levels of PPARα showed to be decreased in mice subjected to pressure overload, elucidating the metabolic substrate switch that characterizes end stage heart failure. In this respect, although MEK1 has been shown to be activated in mice hearts in response to acute pressure overload stimulation induced by aortic banding [27], diminished PPARα activity in these hearts is probably due to another mechanism that is unrelated to enhanced MEK1 activity.

In contrast, mice subjected to increased workload by voluntary running-wheel exercise showed no significant differences in cardiac PPARα levels compared to untrained mice. Using voluntary running-wheel exercise as a functional in vivo model to stimulate cardiac MEK1 activation, we show that MEK1 signaling inhibits the transcriptional activity of PPARα. Interestingly, MEK1 transgenic mouse lines showed a mild concentric hypertrophy with thicker septum and left ventricular posterior wall with very few signs of histopathology or interstitial fibrosis [13]. Moreover, echocardiography demonstrated an enhanced contractile performance in these mice, suggesting compensated cardiac hypertrophy as seen in physiological hypertrophy. Indeed, exercise induced hypertrophy resulted in activation of the downstream effectors ERK1/2 indicating increased MEK1 activity, indicating that the preserved cardiac function, such as seen in MEK1 transgenic mouse lines, is likely to be PPARα independent. Thus, we present here a novel mechanism of downregulation of PPARα activity through MEK1 induced redistribution from the nucleus to the cytosol. This ERK1/2 independent nuclear shuttling of PPARα by MEK1 provides an attractive explanation for the metabolic switch during cardiac hypertrophy and will lead to new insights into the different mechanisms between pathological and physiological hypertrophy.

## Materials and Methods

### Cage-wheel Exercise

This study was carried out in strict accordance with the recommendations in the Guide for the Care and Use of Laboratory Animals of the National Institutes of Health. The protocol was approved by the Committee on the Ethics of Animal Experiments of the University Medical Center Utrecht, Utrecht, the Netherlands (Permit Number: 2009.II.03.024). Male C57BL6/J mice were subjected to voluntary cage wheel exercise as described [32], [33]. Briefly, individual animals were individually housed in a cage equipped with an 11.5-cm-diameter running wheel with a 5.0-cm-wide running surface equipped with a digital magnetic counter activated by wheel rotation. Twice a week, mice received an intraperitoneal injection of U0126 (Cell Signaling), 40 µg/kg, or vehicle alone. Daily exercise values for time and distance run were recorded for individual exercised animals throughout the duration of the exercise period (4 weeks).

### Cell Culture

Low passage HEK293 and NKL-TAg cells [18] were cultured in Dulbecco’s modified Eagle’s medium (Invitrogen) supplemented with 10% fetal bovine serum.

### Transient Transfections and Luciferase Assays

Transfections were performed in 24-well plates (1×10^4^ cells/well). After 24 hours, transient transfections were performed as described [34], [35] with FuGENE 6 reagent as per the manufacturer’s recommendations. After 8 hours, cells were refreshed with serum-free medium with or without the respective stimuli with Wy-14643 (1 µM) or U0126 (5 µM) for 2 hours. After 24 hours, cells were then harvested and lysates were analyzed for firefly luciferase expression. In brief, 20 µl aliquots of cell lysates were mixed with 40 µl of luciferase reagent buffer (Promega Corp) and luminescence of the samples was integrated over a period of 10 seconds in a LUMAC Biocounter M1500P (Landgraaf). To assess transfection efficiency, a SV40 promoter driven Renilla luciferase vector was co-transfected and measured using the Dual Luciferase Assay (Promega Corp). For siRNA experiments, cells were transfected with controle siRNAs or siRNAs specific for ERK1/2 (Ambion) in a final concentration of 10 nM, using Oligofectamine (Invitrogen). Twenty-four hours after transfection, cells were washed in PBS and further treated as described.

### Vector Construction

The reporter plasmid containing the *muscle type carnitine palmitoyltransferase 1b* promoter (*cpt1a* or mCPT1) linked to firefly luciferase (mCPT1-luc) was described previously [36]. Substitution of Serine 12, 21, 76 with Alanine in pcDNA4/TO-PPARα was engineered by PCR-mediated site-directed mutagenesis (Stratagene). Expression vectors encoding an N-terminal fusion between a long-lived GFP protein and mouse PPARα or PPARδ were generated by cloning either full length cDNA into vector pAcGFP1-N1 (Clontech) as HindIII/SacII inserts.

### Immunoprecipitations, Western Blotting

Immunoprecipitation assays were performed as described previously [34] either after transfection of pCDNA3.1-PPARα and/or pCDNA3.1-MEK1, using FuGene6 reagent [37] followed by purification by immunoprecipitation of polyclonal PPARα antibody (Santa Cruz) or MEK1 antibody (Cell signaling) with the Catch and Release kit (Upstate). Proteins were extracted using cell lysis buffer (20 mM Tris pH 8.0, 150 mM NaCl, 1 mM EDTA, 1 mM EGTA, 1% Triton X-100) supplemented with a protease inhibitor cocktail (Complete Mini, Roche). Western blotting was performed as described previously [38], [39]. Protein intensity was quantified using ImageJ.

#### Quantitative RT-PCR

One microgram of total RNA was used as template for Superscript reverse transcriptase II (Promega). For real time-PCR, a BioRad iCycler (Biorad) and SYBR Green was used in combination with specific primer sets designed to detect transcripts (primer sequences available upon request).

### Nuclear Extract Preparation

Cells were washed with ice-cold PBS, scraped into 5 ml of PBS and pelleted by centrifugation at 1500 rpm for 10 min at 4°C. Cell pellets were washed with buffer A (10 mM Tris-HCl, pH 7.6, 1.5 mM MgCl_2_, 10 mM KCl, supplemented with 2 mM DTT, 0.4 mM PMSF, 2 µg/ml leupeptin, 2 µg/ml aprotinin, 2 µg/ml pepstatin, and 1 mM Na_3_VO_4_), resuspended in buffer A, and incubated on ice for 10 min. Nuclei were pelleted at 3000 rpm for 10 min and resuspended in buffer C (0.42 M KCl, 20 mM Tris-HCl, pH 7.8, 20% (v/v) glycerol, 1.5 mM MgCl_2_) supplemented with 2 mM DTT, 0.4 mM PMSF, 2 µg/ml leupeptin, 2 µg/ml aprotinin, 2 µg/ml pepstatin, and 1 mM Na_3_VO_4_. Nuclear proteins were extracted by stirring at 4°C for 30 min. After centrifugation at 13,500 rpm for 30 min, the supernatant was dialyzed against buffer Z-100 (25 mM Tris-HCl, pH 7.6, 0.2 mM EDTA, 20% (v/v) glycerol, 2 mM DTT, 0.4 mM PMSF, 1 mM Na_3_VO_4_, 100 mM KCl) at 4°C. The dialysate was clarified by centrifugation at 13,500 rpm for 30 min at 4°C and designated as crude nuclear extract.

### Immunofluorescence

Paraformaldehyde-fixed HEK293 cells were permealized with 0.2% Triton X-100 in PBS for 5 minutes. Primary monoclonal MEK1 antibody (Cell signaling; 1∶500) and secondary monoclonal anti-mouse Texas-Red (Santa Cruz; 1∶500) antibodies were diluted using 1% BSA in TBS and incubations were carried out at room temperature for 1 hour. Cells were washed 3 times with PBS for 5 minutes, mounted with coverslips in Vectashield mounting medium H-1000 (vector Laboratories, Inc., CA USA), and analyzed by immunofluorescence microscopy using a Zeiss LSM 510 META instrument [40] and nuclear GFP intensity was quantified using ImageJ. Nuclei were counterstained with DAPI.

### Statistical Analysis

Results are presented as means ± SEM. Statistical analyses were performed using INSTAT 3.0 software (GraphPad, San Diego) and Student’s t-test or ANOVA followed by Tukey’s post-test when appropriate. Statistical significance was accepted at a P value <0.05.
